# Rapid identification platform of spores based on Raman spectroscopy

**DOI:** 10.1016/j.fochx.2025.102844

**Published:** 2025-07-28

**Authors:** Longgang Yan, Miaoyun Li, Yaodi Zhu, Yangyang Ma, Lijun Zhao, Lingxia Sun, Gaiming Zhao, Dong Liang

**Affiliations:** a College of Food Science and Technology, Henan Agricultural University, Zhengzhou 450002, China; b International Joint Laboratory of Meat Processing and Safety in Henan Province, Henan Agricultural University, Zhengzhou 450002, China

**Keywords:** Spore contamination, Food safety, Raman spectroscopy, Spore identification, Python-based platform, Microbial contaminants

## Abstract

The food processing stage facilitates the survival of spores from microorganisms such as *Clostridium perfringens* and *Bacillus* species, thereby posing food safety risks. However, current methods for detecting and classifying spore contamination in food are slow, inefficient, and unsuitable for rapid comparison. This method utilizes Raman spectroscopy, combined with a Python-based platform for comparison and analysis. Six common *Clostridium* and *Bacillus* spores were analyzed, with distinct Raman spectral peaks identified at 838 cm^−1^, 895 cm^−1^, 1052 cm^−1^, 1200 cm^−1^, 1400 cm^−1^, 1577 cm^−1^, 1666 cm^−1^, 1722 cm^−1^, 2970 cm^−1^, and 3000 cm^−1^. The platform was validated using bone gelatin products, successfully identifying *Clostridium perfringens*, *Bacillus subtilis*, *Bacillus cereus*, and *Bacillus thuringiensis* spores. This method enables faster and more efficient spore detection and classification, providing valuable technical support for improving food safety and contamination control strategies.

## Highlights

• Differential analysis of spore types using Raman spectroscopy.
• Developing a method for identifying spore categories based on data comparison using Python.
• Rapid identification of spore contamination in bone meal products.

## 1 Introduction

The global food industry has made significant strides in processing technologies, yet microbial contamination remains a major challenge. Particularly concerning spores, which are produced by bacteria and fungi and can survive harsh conditions such as heat and chemicals, posing risks to food safety and human health (Barbosa, 2025; Michel et al., 2024). Spores from pathogens like *Clostridium perfringens* and *Bacillus* species can germinate in favorable conditions, leading to foodborne illnesses. Efficient detection and identification of these spores are crucial to maintaining food safety (Gao et al., 2024; Pahalagedara et al., 2024). Both *Clostridium* and *Bacillus* species are found in a wide range of foods, with contamination levels varying depending on factors such as food type, processing methods, and storage conditions. *Clostridial* spores, including those from *Clostridium perfringens*, *Clostridium difficile*, and *Clostridium sporogenes*, are frequently found in a variety of food products, particularly animal-based products, canned foods, and ready-to-eat meals. These spores are of major concern due to their ability to survive cooking temperatures and germinate in the intestines upon ingestion, leading to foodborne illness (Boer, de Boer et al., 2011; Issimov et al., 2022). *Bacillus* species, including *Bacillus cereus*, *Bacillus thuringiensis*, and *Bacillus subtilis*, are widespread in both plant- and animal-based foods. These spores are known for their resilience to heat and are commonly found in products such as rice, dairy, flour, and dried foods (Rahnama et al., 2023). In 2006, 19 strains of *Bacillus thuringiensis* were isolated from various food samples. This suggests that *Bacillus thuringiensis* may enter food and beverages through residues from the use of agricultural biopesticides, particularly in samples such as green tea beverages and pasteurized full-fat milk. This finding highlights the potential risk of *Bacillus thuringiensis* contamination in food products due to the residual presence of biopesticides (Zhou et al., 2008).

Traditional microbiological culture methods for identifying microbial contamination in food have significant drawbacks. These techniques are time-consuming, requiring long incubation periods and complex procedures that hinder rapid detection and classification. Additionally, they are limited in differentiating closely related species or strains based solely on phenotypic characteristics. As a result, there is an urgent need for faster, more reliable methods that can provide real-time detection and precise identification of spore contaminants in food products (Garavelli et al., 2019; Kuppusamy et al., 2024). Molecular biology-based techniques have been extensively applied in the field of microbial detection. Methods such as Polymerase Chain Reaction (PCR), Quantitative PCR (qPCR), and Loop-mediated Isothermal Amplification (LAMP) are widely used for the rapid and sensitive identification of microorganisms. The PCR-DGGE (Polymerase Chain Reaction-Denaturing Gradient Gel Electrophoresis) technique represents an innovative molecular tool that holds significant potential for the detection of plant pathogens (Sheikha & Ray, 2014). It is capable of effectively identifying and characterizing pathogens within complex plant samples. However, its widespread application is currently limited by the complexity of the procedure and the relatively high costs associated with its implementation. While these techniques are highly effective in detecting specific pathogens, they often require complex sample preparation, skilled operators, and advanced laboratory infrastructure, which can limit their applicability in field settings or for real-time monitoring. In recent years, advances in spectroscopic techniques, particularly Raman spectroscopy, have opened up new possibilities for the rapid and non-destructive identification of microbial contamination. Raman spectroscopy is based on the scattering of monochromatic light by molecules, and it provides detailed information about the molecular vibrations of chemical bonds (Wu et al., 2023). Raman spectroscopy offers a distinct advantage in spore analysis, as the spectral signatures of different spores vary due to differences in their biochemical composition, enabling the differentiation of spore types. This spectral fingerprinting capability enables the rapid identification and classification of spores based on their Raman spectra, without the need for complex sample preparation or lengthy incubation periods (Wu et al., 2023; Zhu et al., 2024). Despite the promise of Raman spectroscopy, current methods for detecting and identifying spore contamination in food have not yet achieved the level of efficiency and speed required for routine use in the food industry (Xiao et al., 2023). A major limitation is the absence of automated platforms for the rapid comparison and classification of spore contaminants in food samples. Manual interpretation of Raman spectra is time-intensive and error-prone, particularly in complex food matrices containing diverse microbial contaminants. Hence, there is an urgent need for advanced systems that can automate spore identification and classification through Raman spectroscopy.

This study investigates a Python-based platform that integrates Differential Interference Contrast (DIC) microscopy and Raman spectroscopy to enhance the detection and classification of spores. The DIC microscopy technique is employed to precisely identify target spores within samples. Subsequently, Raman spectral information is collected from these samples to obtain comprehensive spectral data. By leveraging the distinct spectral characteristics of each type of spore, the platform utilizes Python's capabilities for rapid data acquisition, comparison, and batch processing. This programmatic approach enables the efficient screening and identification of large datasets, thereby reducing the complexity of manual comparison and enhancing the efficiency of data analysis. In practical applications, the number of samples and the quantity of spores within each sample can be extremely large, with datasets often comprising hundreds or even thousands of entries. Manual interpretation of Raman spectra requires individuals to manually identify and mark the characteristic peaks of spores, accurately memorize the specific peak positions for each type of spore, and compare spectral information line by line. This process is labor-intensive and time-consuming, making it impractical for rapid feedback on contamination status. By employing the method presented in this study, batch processing and comparison of data can be achieved, allowing for simultaneous data acquisition, comparison, and classification. The full utilization of computational power enables the platform to perform rapid and accurate identification, significantly improving the efficiency and feasibility of spore detection and classification in large-scale applications.

## 2 Material and methods

### 2.1 Experimental materials

Sample types: ready-to-eat soups, defatted bone extracts, bone soups, bone stocks, essence pastes. Research for commercially available osteopontin products and laboratory osteopontin products, the main product sources Zhengzhou (Henan Province), Hebi (Henan Province), Zhumadian (Henan Province), Kaifeng (Henan Province), Fushun (Liaoning Province), Guangzhou (Guangdong Province) and other areas, different batches of samples, a total of 51 samples were taken (Table 1). Five distinct types of bone broth products were randomly purchased from the market, covering various brands and different batches available for sale. To ensure diversity in batch numbers and brands, we selected well-established brands with a stable sales record. Ultimately, 51 samples meeting the experimental requirements were obtained. A random sampling standard was applied to purchase samples from different batches, and each sample was subjected to a five-point sampling process to ensure representativeness.

**Table 1:** Bone extracts Product Label.

Sample Number	Sample Name	Sample Number	Sample Name
1	Ready-to-eat A Batch 1	27	Bone soup B Batch 1
2	Ready-to-eat A Batch 2	28	Bone soup B Batch 2
3	Ready-to-eat A Batch 3	29	Bone soup B Batch 3
4	Ready-to-eat B Batch 1	30	Bone soup B Batch 4
5	Ready-to-eat B Batch 2	31	Bone soup B Batch 5
6	Ready-to-eat B Batch 3	32	Bone stock A Batch 1
7	Defatted bone extract A Batch 1	33	Bone stock A Batch 2
8	Defatted bone extract A Batch 2	34	Bone stock A Batch 3
9	Defatted bone extract A Batch 3	35	Bone stock A Batch 4
10	Defatted bone extract A Batch 4	36	Bone stock A Batch 5
11	Defatted bone extract A Batch 5	37	Bone stock B Batch 1
12	Defatted bone extract B Batch 1	38	Bone stock B Batch 2
13	Defatted bone extract B Batch 2	39	Bone stock B Batch 3
14	Defatted bone extract B Batch 3	40	Bone stock B Batch 4
15	Defatted bone extract B Batch 4	41	Bone stock B Batch 5
16	Defatted bone extract B Batch 5	42	Essence Paste A Batch 1
17	Defatted bone extract C Batch 1	43	Essence Paste A Batch 2
18	Defatted bone extract C Batch 2	44	Essence Paste A Batch 3
19	Defatted bone extract C Batch 3	45	Essence Paste A Batch 4
20	Defatted bone extract C Batch 4	46	Essence Paste A Batch 5
21	Defatted bone extract C Batch 5	47	Essence Paste B Batch 1
22	Bone soup A Batch 1	48	Essence Paste B Batch 2
23	Bone soup A Batch 2	49	Essence Paste B Batch 3
24	Bone soup A Batch 3	50	Essence Paste B Batch 4
25	Bone soup A Batch 4	51	Essence Paste B Batch 5
26	Bone soup A Batch 5		

Note: “+” indicates detection, “-” indicates not detected.

### 2.2 Culture media for cultivating clostridium species

The media utilized in this study, including Reinforced Clostridial Medium (RCM), Nutrient Agar (NA), Tryptose Sulfite Cycloserine Agar Base (TSC), and Wort Beef Extract Peptone Agar Medium, were all procured from Beijing Land Bridge Technology Co., Ltd.

### 2.3 Budding spore isolation and counting

The sample was weighed 25 g in 225 mL saline and prepared into a series of 10^−1^–10^−3^ bacterial suspensions according to the 10-fold dilution method. The bacterial suspension was kept in a water bath at 80 °C for 20 min to kill non-spores, and the number of sproes was counted by phase contrast microscopy by aspirating drops of the suspension on a 25 × 16 hemocytometer plate (the spores were in a white bright state under phase contrast microscopy); 100 μL of the suspension was identified by Raman spectroscopy; 100 μL of the suspension was spread on five media and identified by subsequent incubation for 16S rDNA identification (Li et al., 2024).

### 2.4 Raman spectroscopy budding spore database construction

#### 2.4.1 Spores preparation

##### 2.4.1.1 *Bacillus cereus (B. cereus)* spores

*Bacillus cereus* was obtained from the China General Microbiological Culture Collection Center (CGMCC, Beijing, China). Precultured bacteria were incubated at 37 °C with agitation for an overnight period in 50 mL of Luria-Bertani broth (Merck KGaA, Darmstadt, Germany). Preculture was added to 50 mL of a modified sporulation medium made, which included 8 g of nutrient broth (Difco, BD), 10 μM FeSO_4_·7H_2_O, 2.5 μM CuCl_2_·2H_2_O, 12.5 μM ZnCl_2_, 66 μM MnSO_4_·4H_2_O, 1 mM MgCl_2_·6H_2_O, 5 mM (NH4)_2_SO_4_(Merck KGaA), 2.5 mM Na_2_MoO_4_·2H_2_O (Sigma-Aldrich), 2.5 μM CoCl_2_·6H_2_O (Sigma-Aldrich), and 1 mM Ca(NO_3_)_2·_4H_2_O (Merck KGaA). Filter-sterilized (0.2 mm-pore-size cellulose acetate; Whatman GmbH, Dassel, Germany) Ca(NO3)_2_·4H_2_O, MnSO_4_·4H_2_O, and FeSO_4_·7H_2_O were added to the medium after it had been autoclaved at 121 °C for 20 min. The pH was adjusted to 7.6 before autoclaving, and the pH of the final sporulation media was between 7.1 and 7.4 (Abhyankar et al., 2016).

##### 2.4.1.2 *Bacillus thuringiensis* (*B. thuringiensis*) spores

*Bacillus thuringiensis* was obtained from the China General Microbiological Culture Collection Center (CGMCC, Beijing, China). The aim of this study is to cultivate and purify *Bacillus thuringiensis* (*B. thuringiensis*) spores for subsequent food safety and microbial contamination analysis. Initially, appropriate media are selected, including Nutrient Agar, Luria-Bertani (LB) medium, and a low-nutrient medium to promote spore formation. Prior to inoculation, the bacterial strain is cultured in Tryptic Soy Broth (TSB) until the logarithmic growth phase. The inoculum is then transferred to spore-inducing media and incubated at 28–30 °C with shaking at 150–200 rpm for 48–72 h to stimulate spore formation. Spores are collected by centrifugation and washed with saline to remove impurities. Further purification is achieved through density gradient centrifugation to enhance spore purity. Storage methods include short-term preservation at 4 °C and long-term storage through freeze-drying (Stanford et al., 2016).

##### 2.4.1.3 *Bacillus subtilis (B. subtilis)* spores

*Bacillus subtilis* was obtained from the China General Microbiological Culture Collection Center (CGMCC, Beijing, China). To cultivate *Bacillus subtilis* spores, the bacteria are first grown on Nutrient Agar (NA) or Tryptic Soy Broth (TSB) at 37 °C for 12–18 h to reach the exponential growth phase. The culture is then transferred to a sporulation medium, typically a low-nutrient broth (e.g., 1 % glucose, 0.5 % NaCl, 0.05 % K_2_HPO_4_), and incubated at 30–37 °C for 24–48 h to induce sporulation. After spore formation reaches 90–95 %, the spores are harvested by centrifugation at 5000–7000 ×*g* for 15–20 min. The resulting pellet is washed with sterile water to remove contaminants. Finally, purified spores are stored at 4 °C for short-term use or freeze-dried for long-term storage (Korza & Setlow, 2013).

##### 2.4.1.4 *Clostridium perfringens (C. perfringens)* spores

The wild-type *Clostridium perfringens* strain C1, isolated from vacuum-packed cooked meat, was obtained from the Microbiology Laboratory of Henan Agricultural University (Zhengzhou, China) and identified by whole-genome sequencing through Sangon Biotech Co., Ltd. (Shanghai, China). The strain was stored at −80 °C after being coated on magnetic beads. To culture the bacteria, the strain was streaked on Tryptose Sulfite Cycloserine Agar (TSC) and incubated anaerobically at 37 °C for 24 h in a gas mixture of 10 % H_2_, 10 % CO_2_, and 80 % N_2_. Typical black colonies were transferred to cooked meat medium for 48 h at 37 °C. A 0.1 mL aliquot of the stock culture was then inoculated into freshly prepared fluid thioglycollate medium (FTG) and incubated for 18 h at 37 °C. Afterward, 1.0 mL of the culture was transferred into fresh FTG and incubated for 4 h at 37 °C. Subsequently, a 0.1 mL aliquot was inoculated into Duncan-Strong sporulation medium and incubated for 24 h at 37 °C. The morphology of the culture was observed under an optical microscope after bacterial staining. Once spore conversion reached 95 %, the culture was harvested by centrifugation at 7000 ×*g* for 20 min at 4 °C and washed twice with sterile distilled water to purify the spores (Liang et al., 2023).

##### 2.4.1.5 *Clostridium difficile* (*C. difficile*) spores

*Clostridium difficile* was obtained from the American Type Culture Collection (ATCC). To cultivate *Clostridium difficile* spores, the bacteria are first cultured on Brain Heart Infusion (BHI) agar or Tryptic Soy Agar (TSA) with 15 % sheep blood, or in Tryptic Soy Broth (TSB) or *C. difficile* broth for liquid cultures. The culture is incubated anaerobically at 37 °C for 24–48 h to promote vegetative growth. For sporulation, the culture is transferred to a sporulation medium, such as liver infusion broth or reinforced clostridial broth, and incubated at 37 °C for 48–72 h under anaerobic conditions. Once 90–95 % of the cells have formed spores, spore formation is confirmed microscopically. Spores are then harvested by centrifugation at 5000–7000 ×*g* for 15–20 min and washed to remove residual medium and cells. Finally, the purified spores are stored at 4 °C for short-term use or freeze-dried for long-term storage (Wang et al., 2015).

##### 2.4.1.6 *Clostridium sporogenes* (*C. sporogenes*) spores

*Clostridium sporogenes* was obtained from the China General Microbiological Culture Collection Center (CGMCC, Beijing, China). To cultivate *C. sporogenes* spores, the bacteria are first cultured on Brain Heart Infusion (BHI) agar or Tryptic Soy Agar (TSA) with 5 % sheep blood, or in liquid Tryptic Soy Broth (TSB) or reinforced clostridial broth. The culture is incubated anaerobically at 37 °C for 24–48 h for vegetative growth. For spore induction, the culture is transferred to a sporulation medium, such as liver infusion broth (LIB) or reinforced clostridial broth (RCB), and incubated at 37 °C for 48–72 h under anaerobic conditions. Sporulation is confirmed microscopically when 90–95 % of the cells form spores. The spores are then harvested by centrifugation at 5000–7000 ×*g* for 15–20 min, washed with sterile water or PBS, and stored at 4 °C for short-term use or freeze-dried for long-term storage. This method ensures high-purity isolation of *C. sporogenes* spores for research and applications (Mah et al., 2009).

#### 2.4.2 Raman spectroscopy feature signal acquisition

Raman spectra were collected using a laser confocal Raman microscope (LabRAM HR Evolution, HORIBA Science, France). A 50 × telephoto microscope was used in the experiments to obtain clearer images of individual cells and the focused laser beam (532 nm, laser power:100 mW). Raman spectra were recorded by an open electrode CCD detector with a spectral resolution of 1 μm. The acquisition time was 15 s, with 2 cumulative acquisitions. Spectra were acquired in the range of 400–3200 cm^−1^. 20 sample points were collected for each sample. The spectral data were normalized using nonlinear least squares (NLS) method.

#### 2.4.3 Strain identification and matching in Python based on eigenvalue ranges

The collected spore spectral data are analyzed by identifying species-specific peaks based on differences in peak positions. These differential peaks are annotated, providing a basis for the classification of spore species. The methodology is outlined as follows:

A. Definition of Characteristic Value Ranges:

A predefined array, referred to as MATCH_ARR, is used to match spectral peaks associated with specific bacterial species. The NAME_DICT dictionary maps bacterial species names to corresponding indices in the characteristic value range array. The parameter MATCH_NUMBER defines the minimum number of characteristic peaks required for a valid match.

B. Reading Exported Spectral Data Files:

The filePath function is employed to recursively access all relevant data files (in.txt format) within a specified directory, each containing the characteristic spectral data for analysis.

C. Identification of Characteristic Values:

The fileRead function extracts the spectral features from the identified files and filters out any values that exceed a predefined threshold (e.g., PEAK_VALUE = 100). The calcType function then compares the extracted values against the predefined ranges in MATCH_ARR. If at least three characteristic values from a file fall within the specified ranges, the bacterial species is identified, and the matching results are recorded.

D. Output of Identification Results:

All matching results are recorded and output to a designated file (e.g., ret.txt) using the fileWrite function for subsequent analysis.

#### 2.4.4 Phase contrast microscopy observation of spore morphology

Observe the spore morphology of the sample under a 40 × phase contrast microscope (Liang et al., 2025).

### 2.5 Statistical analysis

Each treatment had at least three independent replicates with two samples per replicate. Statistical analyses were performed using the mean microbial reduction (expressed as logarithmic values). One-way analysis of variance (ANOVA) was performed using SPSS (version 26.0, Norman H. Nie, CA, USA) to compare differences between groups. *p* < 0.05 was considered significant. Graphs were plotted using Origin 2021 software (Qu et al., 2025).

## 3 Results and discussion

### 3.1 Differential Raman spectroscopy analysis of six types of spores

**Fig. 1:**
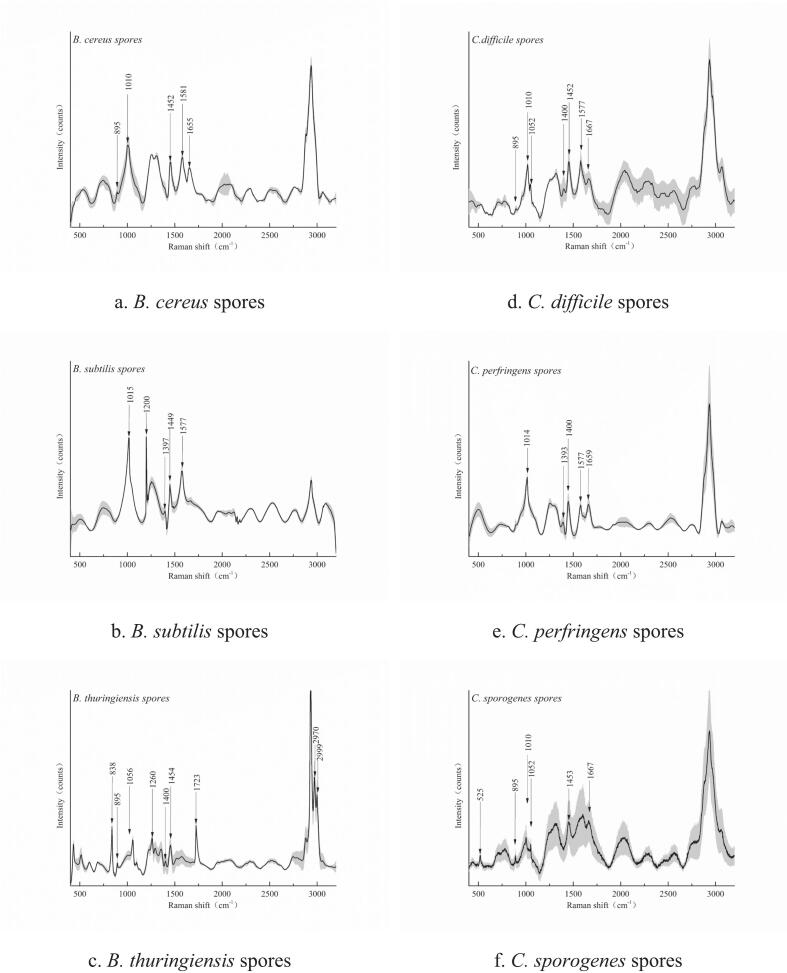
Raman Spectral Difference Feature Map of *Clostridium* and *Bacillus* Spores.

The spectral characteristics of different *Bacillus* species at specific wavelengths (Fig. 1) reveal distinct peaks corresponding to various chemical bonds or molecular structures. For instance, a peak at 838.44 cm^−1^ corresponds to C—N stretching vibrations, suggesting the potential presence of proteins and nucleic acids (Zaytseva et al., 2024). A peak at 895.43 cm^−1^ is associated with C-O-C stretching vibrations, which may indicate the presence of sugars and ester compounds. The peak at 1010.49 cm^−1^ is linked to carbohydrate, C—C, C—O, and C-OH deformation vibrations, implying the presence of polysaccharides and other carbohydrates (Costa et al., 2024). At 1200.68 cm^−1^, the peak corresponds to C-C_6_H_5_ stretching, along with phenylalanine and tryptophan, indicating the likely presence of proteins. A peak at 1256.32 cm^−1^ represents N—H, C—N, and amide III vibrations, suggesting the possible presence of proteins and peptides. The 1399.92 cm^−1^ peak corresponds to symmetric stretching of COO-, indicating the presence of carboxylic acids and amino acids. At 1577.22 cm^−1^, a peak is observed that relates to extracellular polymeric substances, suggesting molecules present in the cell wall and extracellular matrix (Muthuraman et al., 2025). The 1666.73 cm^−1^ peak is associated with amide I stretching, pointing to the presence of proteins (Pan et al., 2025). A peak at 1722.54 cm^−1^ corresponds to C=O stretching, which may indicate the presence of carboxylic acids, ketones, and aldehydes. The peak at 2970.11 cm^−1^ is related to C—H stretching, suggesting the presence of aliphatic compounds (Sah et al., 2025). Finally, a peak at 2999.12 cm^−1^ corresponds to O—H and N—H stretching, implying the potential presence of water, alcohols, and amines (Sung et al., 2024).

The different *Bacillus* species, including *B. thuringiensis* spores, *B. cereus* spores, *B. subtilis* spores, *C. perfringens* spores, *C. difficile* spores, and *C. botulinum* spores (Table 2), exhibit characteristic absorption peaks across multiple wavelengths (Lai et al., 2024). These peaks suggest the presence of a variety of compounds, such as C—N bonds, C-O-C bonds, carbohydrates, phenylalanine, tryptophan, COO- groups, C=O bonds, C—H bonds, and O—H, N—H bonds. These characteristic absorption peaks not only reveal the chemical diversity of the bacteria but also highlight their similarities and differences in certain compounds. Such differences may be related to the bacteria's metabolic pathways, cellular structures, and functional characteristics.

**Table 2:** Typical Raman Spectral Difference Peaks between *Clostridial* and *Bacillus* Spores.

Wavenumber (cm^−1^)	*B. thuringiensis* spores	*C. sporogenes* spores	*B. cereus* spores	*C. perfringens* spores	*B. subtilis* spores	*C. difficile* spores
838.44	+	−	−	−	−	−
895.43	+	+	−	−	+	+
1010.49	−	+	+	+	+	+
1051.94	+	−	−	−	+	+
1200.68	−	−	+	−	−	−
1256.32	+	+	−	−	−	−
1399.92	+	−	+	+	+	−
1577.22	−	+	+	+	+	+
1666.73	−	+	−	+	−	+
1722.54	+	−	−	−	−	−
2970.11	+	−	−	−	−	−
2999.12	+	−	−	−	−	−

Note: “+” indicates detection, “-” indicates not detected.

In conclusion, these spectral features provide critical insights into the biological characteristics of these bacteria. With further research, these spectral characteristics can be used to distinguish between different *Bacillus* species and serve as valuable differential data points for developing a Python-based comparative platform for microbial analysis.

### 3.2 Python-based comparison platform development

In this study, the identification and classification of different bacterial species, based on their spectral characteristics, were carried out using a Python script (Wang et al., 2025). The primary goal of this script was to match the spectral peaks extracted from various files with known peak ranges associated with specific *Bacillus* species. The analysis follows a systematic approach to process spectral data, compare it with predefined characteristic values, and classify the bacterial species accordingly.

The program uses specific peak value ranges, defined in the array `MATCH_ARR`, which represent spectral features corresponding to different molecular structures. These spectral features are indicative of the chemical bonds and functional groups present in the bacteria. The bacterial species are linked to specific indices in the `NAME_DICT`, which outlines which peak values (from the `MATCH_ARR` ranges) correspond to which species. For example, the species *C. botulinum* spores are associated with the first, second, and seventh characteristic peaks, while *B. thuringiensis* spores are linked to a broader range of peaks (Van Uffelen et al., 2024).

The script works by recursively searching through a given directory for files that contain spectral data. It reads each file, extracting the peak values that exceed a threshold of 100 (as defined by `PEAK_VALUE`), which is crucial for identifying meaningful spectral peaks (Wang et al., 2025). Once these values are extracted, they are compared with the predefined characteristic values for each bacterial species. The goal is to identify matches based on the presence of at least three matching spectral features, as set by the `MATCH_NUMBER` threshold.

Once the comparison is complete, the script classifies the spectral data based on the number of matching peaks and records the result. If a match is found, the file path and the corresponding bacterial species are added to the `result_list`, and the matching result is printed. If no match is found, the program prints that the species could not be identified from the data. Finally, the results are written to an output file for further analysis (Guerrero-Egido et al., 2024).

The results of this analysis show that the program can successfully identify the species present in the spectral data by matching peaks to known values. Each bacterial species was associated with a unique combination of spectral features, which allows for reliable classification. The program's effectiveness lies in its ability to accurately match spectral peaks from unknown data with predefined characteristic peaks, offering a robust approach for bacterial identification. The use of a minimum match threshold ensures that only reliable matches are considered, reducing the likelihood of misclassification.

Furthermore, this approach could be expanded for use in a broader range of microbial identification tasks (Zaatry et al., 2024). By refining the peak value ranges and adjusting the match threshold, the system could potentially be applied to different bacterial species or other microorganisms, enhancing its utility in microbial research and diagnostics.

**Fig. 2:**
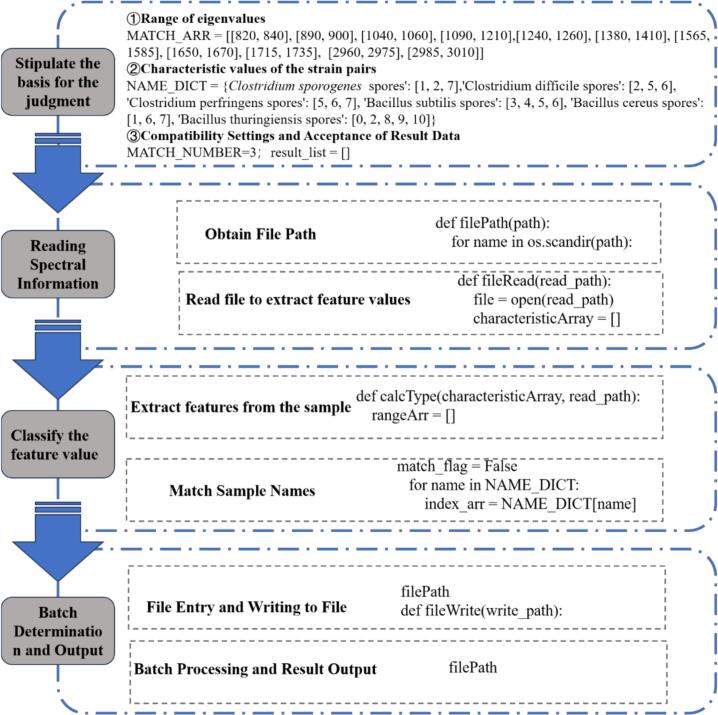
Schematic diagram of Python data scraping and comparison code.

In summary, the implementation of this spectral peak matching system provides a promising tool for the rapid identification of bacterial species based on their spectral data. With further refinement and testing, it could serve as a valuable asset in microbiological studies, offering a high-throughput, automated method for species identification and classification (See Fig. 2).

### 3.3 The spore contamination situation in bone extracts products

The table presents a comparison of spore counts (in logarithmic Colony Forming Units per gram, log CFU/g) across various samples, with each entry accompanied by the associated standard deviation, indicating the variability within each sample group. This data is crucial for evaluating the microbial load in different food products and could serve as an important reference for quality control, safety assessments, and microbiological research.

The samples are grouped into categories such as ready-to-eat products, defatted bone extracts, bone soups, bone stocks, and essence pastes. For instance, the ready-to-eat products (Ready-to-eat A and Ready-to-eat B) exhibit relatively low spore counts of 1.16 ± 0.05 and 0.22 ± 0.09 log CFU/g, respectively. These lower spore counts may indicate a relatively higher degree of microbial control or better preservation techniques in ready-to-eat food items, suggesting these products are less likely to pose a microbial risk compared to other categories.

In contrast, the defatted bone extracts samples, such as Defatted bone extract A, Defatted bone extract B, and Defatted bone extract C, show much higher spore counts, with values ranging from 7.18 ± 0.05 to 7.25 ± 0.04 log CFU/g. These higher counts suggest that defatted bone extracts products may be more prone to contamination or may have undergone less stringent sterilization or preservation methods, warranting additional attention for microbial safety during production and storage (Pinto et al., 2025).

The bone soups (Bone soup A and Bone soup B) and bone stocks (Bone stock A and Bone stock B) also show relatively high spore counts, ranging from 5.34 ± 0.10 to 6.55 ± 0.07 log CFU/g. These values suggest a moderate microbial load in these types of products. The difference in spore counts between the bone soups and bone stocks may indicate variations in processing or ingredient composition, with potential implications for their shelf life and microbial stability (Juneja et al., 2024).

Essence paste samples, such as Essence Paste A and Essence Paste B, present lower spore counts compared to defatted bone extracts and soups, with values of 5.82 ± 0.15 and 5.76 ± 0.09 log CFU/g, respectively. Although these counts are still relatively high, they indicate a more controlled microbial environment compared to the defatted bone extracts and soups. This could be attributed to differences in the paste's formulation, preparation, or packaging, which may offer some degree of protection against microbial contamination (Snyder et al., 2024).

The samples were collected in multiple batches, and factors such as production differences across batches, variations in processing temperatures, and raw material batch discrepancies may contribute to inconsistencies in spore contamination levels among the samples. Therefore, variance analysis was conducted for different batches of the same sample. The error values for the samples were all below 0.21, indicating that the error was relatively small and the variability across different batches was minimal. This suggests that the processing steps are relatively stable (Table 3).

**Table 3:** Number of Spores in Samples.

Sample Name	Spore Count (log CFU/g)	Sample Name	Spore Count (log CFU/g)
Ready-to-eat A	1.16 ± 0.05	Bone soup A	6.39 ± 0.21
Ready-to-eat B	0.22 ± 0.09	Bone soup B	6.34 ± 0.09
Defatted bone extract A	7.18 ± 0.05	Bone stock A	6.55 ± 0.07
Defatted bone extract B	7.25 ± 0.03	Bone stock B	5.34 ± 0.10
Defatted bone extract C	7.25 ± 0.04	Essence Paste A	5.82 ± 0.15
		Essence Paste B	5.76 ± 0.09

In conclusion, the spore count data provides valuable insights into the microbial contamination levels of different food samples. The higher spore counts observed in defatted bone extracts and soup products highlight the need for improved microbial control measures in these categories, while the lower counts in ready-to-eat products and essence pastes may suggest more effective preservation methods. The standard deviations indicate that there is some variability within each sample group, which could be reflective of differences in batch processing or storage conditions. This information is critical for ensuring food safety, optimizing production processes, and guiding future research on microbial contamination in food products.

### 3.4 The Python-based comparison platform determines the types of spore contamination in bone extracts products

Using the Python-based comparison platform, rapid data reading and species identification of spore contamination in samples were performed, clearly revealing the spore contamination situation in different samples. The primary contaminating spores identified were *C. perfringens*, *B. subtilis*, *B. cereus*, and *B. thuringiensis*, which may impact food safety and quality.

*C. perfringens*, a well-known pathogen associated with foodborne illness, appears consistently across a range of food types and batches, including ready-to-eat meals (*Ready-to-eat A* and *Ready-to-eat B*), defatted bone extracts (*Defatted bone extract A*, *Defatted bone extract B*, and *Defatted bone extract C*), bone soups (*Bone soup A* and *Bone soup B*), and essence pastes (*Essence Paste A* and *Essence Paste B*). The bacterium is notably present in nearly all batches, emphasizing its widespread contamination potential. Its recurrent presence in food samples highlights the need for stringent microbial monitoring to mitigate the risk of foodborne outbreaks.

*B. subtilis*, a common environmental contaminant, is detected across various batches of food, including ready-to-eat meals, defatted bone extracts, bone soups, and essence pastes. Its presence suggests that contamination likely occurs during the production or storage phases, typically from environmental sources such as soil or air.

*B. cereus*, another pathogen that can cause food poisoning, is also widespread across multiple batches. Found in both soups and defatted bone extracts, its presence is concerning for food safety, particularly in improperly stored or inadequately processed products, as it is known to cause gastrointestinal illnesses.

*B. thuringiensis*, an insecticidal bacterium, is present in several batches, including ready-to-eat meals, defatted bone extracts, bone soups, and essence pastes. While not harmful to humans, its presence may indicate contamination from agricultural environments where this bacterium is used as a biopesticide. The detection of *B. thuringiensis* across a variety of products suggests that agricultural contamination could be a potential source of microbial presence in these food categories, especially for products exposed to external environmental factors during production.

The data reveals that multiple bacterial species are present across a wide range of food samples, suggesting various sources and stages of contamination. Of particular concern is the high frequency of *C. perfringens*, a known foodborne pathogen that poses a significant risk to human health, particularly when found in ready-to-eat meals and soups that are consumed without further cooking. This emphasizes the importance of thorough testing and preventive measures in ensuring food safety (Table 4).

**Table 4:** The Python Spectral Data Comparison Platform Determines the Spore Contamination Situation.

Sample Name	*C. perfringens* spores	*B. subtilis* spores	*B. cereus* spores	*B. thuringiensis* spores	*C. difficile* spores	*C. sporogenes* spores
Ready-to-eat A Batch 1	+	−	−	+	−	−
Ready-to-eat A Batch 2	−	+	−	−	−	−
Ready-to-eat A Batch 3	+	−	+	−	−	−
Ready-to-eat B Batch 1	−	+	−	−	−	−
Ready-to-eat B Batch 2	+	+	−	−	−	−
Ready-to-eat B Batch 3	−	−	+	+	−	−
Defatted bone extract A Batch 1	+	+	−	−	−	−
Defatted bone extract A Batch 2	+	−	+	−	−	−
Defatted bone extract A Batch 3	+	−	−	+	−	−
Defatted bone extract A Batch 4	+	−	+	+	−	−
Defatted bone extract A Batch 5	+	−	−	−	−	−
Defatted bone extract B Batch 1	+	+	+	+	−	−
Defatted bone extract B Batch 2	+	+	−	−	−	−
Defatted bone extract B Batch 3	+	−	+	−	−	−
Defatted bone extract B Batch 4	+	−	−	−	−	−
Defatted bone extract B Batch 5	+	−	−	+	−	−
Defatted bone extract C Batch 1	+	−	+	−	−	−
Defatted bone extract C Batch 2	+	+	−	−	−	−
Defatted bone extract C Batch 3	+	−	−	+	−	−
Defatted bone extract C Batch 4	+	−	+	−	−	−
Defatted bone extract C Batch 5	+	+	−	−	−	−
Bone soup A Batch 1	+	−	−	+	−	−
Bone soup A Batch 2	+	+	−	−	−	−
Bone soup A Batch 3	+	−	−	+	−	−
Bone soup A Batch 4	+	−	+	−	−	−
Bone soup A Batch 5	+	+	−	+	−	−
Bone soup B Batch 1	+	−	+	−	−	−
Bone soup B Batch 2	+	+	−	−	−	−
Bone soup B Batch 3	+	+	−	+	−	−
Bone soup B Batch 4	+	−	+	−	−	−
Bone soup B Batch 5	+	−	−	−	−	−
Bone stock A Batch 1	+	−	+	+	−	−
Bone stock A Batch 2	+	+	−	+	−	−
Bone stock A Batch 3	+	−	−	+	−	−
Bone stock A Batch 4	+	−	+	+	−	−
Bone stock A Batch 5	+	+	+	+	−	−
Bone stock B Batch 1	+	+	−	−	−	−
Bone stock B Batch 2	+	−	+	−	−	−
Bone stock B Batch 3	+	−	−	−	−	−
Bone stock B Batch 4	+	+	+	−	−	−
Bone stock B Batch 5	+	−	+	−	−	−
Essence Paste A Batch 1	+	−	+	−	−	−
Essence Paste A Batch 2	+	+	−	+	−	−
Essence Paste A Batch 3	+	+	+	−	−	−
Essence Paste A Batch 4	+	−	+	−	−	−
Essence Paste A Batch 5	+	−	−	+	−	−
Essence Paste B Batch 1	+	+	−	+	−	−
Essence Paste B Batch 2	+	−	+	+	−	−
Essence Paste B Batch 3	+	−	+	−	−	−
Essence Paste B Batch 4	+	−	+	+	−	−
Essence Paste B Batch 5	+	+	+	+	−	−

Note: “+” indicates detection, “-” indicates not detected.

The results from this analysis highlight the complexity of microbial contamination in food products and underscore the necessity for comprehensive food safety protocols that address the risks posed by various bacterial species. The presence of *C. perfringens*, *B. subtilis*, *B. cereus*, and *B. thuringiensis* in multiple food categories emphasizes the importance of targeted monitoring at every stage of food production—from raw material sourcing and processing to storage and distribution. Further research and validation techniques are necessary to better understand the specific sources of contamination and to develop more effective strategies for microbial control and prevention.

### 3.5 PCR (16S rDNA) verification of spore contamination

In general, *C. perfringens* appears frequently across all batches, with nearly every sample showing its presence, particularly in products such as *Ready-to-eat A*, *Defatted bone extract A*, and *Bone stock A*. This bacterium is commonly associated with foodborne illnesses, suggesting a potential risk factor for these food types (Lovely et al., 2024). Interestingly, it is present in most batches of *Essence Paste* and *Defatted bone extracts* products, with the highest frequency observed in *Defatted bone extract B* and *C* batches, where it is consistently identified alongside other species like *B. cereus* and *B. thuringiensis* (Indriani et al., 2024).

The second bacterium, *Bacillus subtilis*, is also widely distributed across the samples, though it is less consistently detected than *C. perfringens*. Its presence is seen in batches of *Ready-to-eat A* and *Defatted bone extract A*, as well as in *Essence Paste* samples, indicating its common occurrence in these food types. Notably, it is more often present in combination with other bacteria, such as *C. perfringens* or *B. cereus*, reflecting potential contamination from environmental sources.

*Bacillus cereus* is sporadically present in various samples, including *Ready-to-eat A* and *B* batches, and *Defatted bone extract C*. This species is associated with both food spoilage and foodborne illness outbreaks, and its presence suggests possible concerns for food safety in certain batches (Wu et al., 2021). It is found alongside other spore-forming bacteria, which could be indicative of cross-contamination or shared environmental exposure.

Lastly, *B. thuringiensis* appears intermittently across the samples, with notable presence in batches of *Ready-to-eat A*, *Defatted bone extract A*, and *Bone stock B*. Although this bacterium is primarily known for its insecticidal properties, its presence in food products raises questions about contamination from agricultural practices, especially where this bacterium is used in pest control (Salehi Jouzani et al., 2024).

The table directly presents the results of gene detection for the samples based on 16S rDNA, as analyzed through the Python platform. The analysis reveals that contamination in the samples is primarily concentrated in *C. perfringens*, *B. subtilis*, *B. cereus*, and *B. thuringiensis*. We performed rapid comparisons of each sample group using the Python platform, identifying the specific spore species responsible for contamination. The 16S rDNA gene detection was then used to validate the identified contaminating spores, and the results were consistent, with no discrepancies observed.

The results indicate that 16S rDNA effectively confirms the contamination species in the collected samples. Additionally, when compared with the Raman spectroscopy-based classification provided by the Python platform, it further supports the accuracy of the rapid comparison and screening platform. This demonstrates that the Python platform can efficiently identify the species of contaminating spores in the samples. Specifically, for bone broth products, the contamination is primarily attributed to *C. perfringens*, which is introduced either through the environment or raw materials, and thus, targeted control measures can be implemented to address this contamination (Table 5).

**Table 5:** 16S rDNA Identification Comparison of Spore Contamination Situation.

Sample Name	Spore Name	Sample Name	Spore Name
Ready-to-eat A Batch 1	*C. perfringens* *B. thuringiensis*	Bone soup B Batch 1	*C. perfringens* *B. cereus*
Ready-to-eat A Batch 2	*B. subtilis*	Bone soup B Batch 2	*C. perfringens* *B. subtilis*
Ready-to-eat A Batch 3	*C. perfringens* *B. cereus*	Bone soup B Batch 3	*C. perfringens* *B. subtilis* *B. thuringiensis*
Ready-to-eat B Batch 1	*B. subtilis*	Bone soup B Batch 4	*C. perfringens* *B. cereus*
Ready-to-eat B Batch 2	*C. perfringens* *B. subtilis*	Bone soup B Batch 5	*C. perfringens*
Ready-to-eat B Batch 3	*B. cereus* *B. thuringiensis*	Bone stock A Batch 1	*C. perfringens* *B. cereus* *B. thuringiensis*
Defatted bone extract A Batch 1	*C. perfringens* *B. subtilis*	Bone stock A Batch 2	*C. perfringens* *B. subtilis* *B. thuringiensis*
Defatted bone extract A Batch 2	*C. perfringens* *B. cereus*	Bone stock A Batch 3	*C. perfringens* *B. thuringiensis*
Defatted bone extract A Batch 3	*C. perfringens* *B. thuringiensis*	Bone stock A Batch 4	*C. perfringens* *B. cereus* *B. thuringiensis*
Defatted bone extract A Batch 4	*C. perfringens* *B. cereus* *B. thuringiensis*	Bone stock A Batch 5	*C. perfringens* *B. subtilis* *B. cereus* *B. thuringiensis*
Defatted bone extract A Batch 5	*C. perfringens*	Bone stock B Batch 1	*C. perfringens* *B. subtilis*
Defatted bone extract B Batch 1	*C. perfringens* *B. cereus* *B. thuringiensis*	Bone stock B Batch 2	*C. perfringens* *B. cereus*
Defatted bone extract B Batch 2	*C. perfringens* *B. subtilis*	Bone stock B Batch 3	*C. perfringens*
Defatted bone extract B Batch 3	*C. perfringens*	Bone stock B Batch 4	*C. perfringens* *B. subtilis* *B. cereus*
Defatted bone extract B Batch 4	*C. perfringens*	Bone stock B Batch 5	*C. perfringens* *B. cereus*
Defatted bone extract B Batch 5	*C. perfringens* *B. thuringiensis*	Essence Paste A Batch 1	*C. perfringens* *B. cereus*
Defatted bone extract C Batch 1	*C. perfringens* *B. cereus*	Essence Paste A Batch 2	*C. perfringens* *B. subtilis* *B. thuringiensis*
Defatted bone extract C Batch 2	*C. perfringens* *B. subtilis*	Essence Paste A Batch 3	*C. perfringens* *B. subtilis* *B. cereus*
Defatted bone extract C Batch 3	*C. perfringens* *B. cereus*	Essence Paste A Batch 4	*C. perfringens* *B. cereus*
Defatted bone extract C Batch 4	*C. perfringens* *B. thuringiensis*	Essence Paste A Batch 5	*C. perfringens* *B. thuringiensis*
Defatted bone extract C Batch 5	*C. perfringens* *B. subtilis*	Essence Paste B Batch 1	*C. perfringens* *B. subtilis* *B. thuringiensis*
Bone soup A Batch 1	*C. perfringens* *B. thuringiensis*	Essence Paste B Batch 2	*C. perfringens* *B. cereus* *B. thuringiensis*
Bone soup A Batch 2	*C. perfringens* *B. subtilis*	Essence Paste B Batch 3	*C. perfringens* *B. cereus*
Bone soup A Batch 3	*C. perfringens* *B. thuringiensis*	Essence Paste B Batch 4	*C. perfringens* *B. cereus* *B. thuringiensis*
Bone soup A Batch 4	*C. perfringens* *B. cereus*	Essence Paste B Batch 5	*C. perfringens* *B. subtilis* *B. cereus* *B. thuringiensis*
Bone soup A Batch 5	*C. perfringens* *B. subtilis* *B. thuringiensis*		

## 4 Conclusions

In conclusion, this study presents a novel Raman spectroscopic platform for rapid spore contamination detection in food products, emphasizing the effectiveness of this Python-based technology in identifying and classifying spore contaminants, including *C. perfringens*, *B. subtilis*, *B. cereus*, *B. thuringiensis*, *C. difficile*, and *C. sporogenes*. By leveraging distinct spectral signatures, the platform offers a fast, reliable, and scalable solution for microbial monitoring, advancing food safety technology and enabling quick detection of contamination in various food categories.

The findings underscore the varying spore contamination levels across food products, with defatted bone extracts and soup products exhibiting higher contamination rates, suggesting the need for enhanced sterilization or preservation techniques. In contrast, ready-to-eat and essence paste products demonstrate more effective preservation, offering valuable insights into better control measures for food safety. These results highlight the importance of microbiological surveillance in food production and storage processes to ensure public health and product quality.

Furthermore, the study validates the platform's efficacy through comparison with traditional DIC microscopy, confirming its potential as a robust tool for food safety monitoring. This technological advancement contributes significantly to ongoing research aimed at improving food safety practices. By facilitating swift detection and classification of spore contamination, the platform aids food manufacturers, regulatory bodies, and public health organizations in optimizing contamination control strategies, ultimately contributing to the reduction of foodborne illnesses and safeguarding global food supply standards.

## CRediT authorship contribution statement

**Longgang Yan:** Writing – review & editing, Writing – original draft, Data curation, Conceptualization. **Miaoyun Li:** Writing – review & editing, Funding acquisition. **Yaodi Zhu:** Supervision. **Yangyang Ma:** Supervision. **Lijun Zhao:** Supervision. **Lingxia Sun:** Supervision. **Gaiming Zhao:** Supervision, Funding acquisition. **Dong Liang:** Supervision.

## Declaration of competing interest

All authors confirm that there are no financial conflicts related to the technology presented in this study. We have not received any form of economic benefits from the development, application, or promotion of the related technology, nor have we been influenced by any economic factors that might affect the objectivity of our research.

## Data Availability

The raw Raman spectral data supporting the findings of this study are subject to privacy and ethical restrictions and are not publicly available.
